# John Widdicombe’s contribution to respiratory physiology and cough: reminiscences

**DOI:** 10.1186/1745-9974-9-6

**Published:** 2013-03-06

**Authors:** Kian Fan Chung, Jay A Nadel, Giovanni Fontana

**Affiliations:** 1 National Heart & Lung Institute, Imperial College London & Biomedical Research Unit, Royal Brompton Hospital, Dovehouse St, London, SW3 6LY, UK; 2 Cardiovascular Research Institute and Departments of Pulmonary and Critical Care Medicine, Physiology, and Radiology, University of California, San Francisco, California, USA & UCSF School of Medicine, San Francisco, CA, USA; 3 Department of Internal Medicine, University of Florence, Florence, Italy

## Abstract

John Widdicombe has made substantial contributions to respiratory physiology and to the field of cough particularly. He was one of the first to characterise Aδ-myelinated fibres in the airways that could mediate cough and increased breathing. Later on, he initiated the series of international London Cough Symposia that gathered researchers and clinicians on a two-yearly basis to discuss recent results and concepts regarding cough. John Widdicombe was interested in all aspects of cough from the definition to potential new antitussives. This article will focus on his contributions and on his generous personality through reminiscences from three friends.

## Introduction

**Figure 1:**
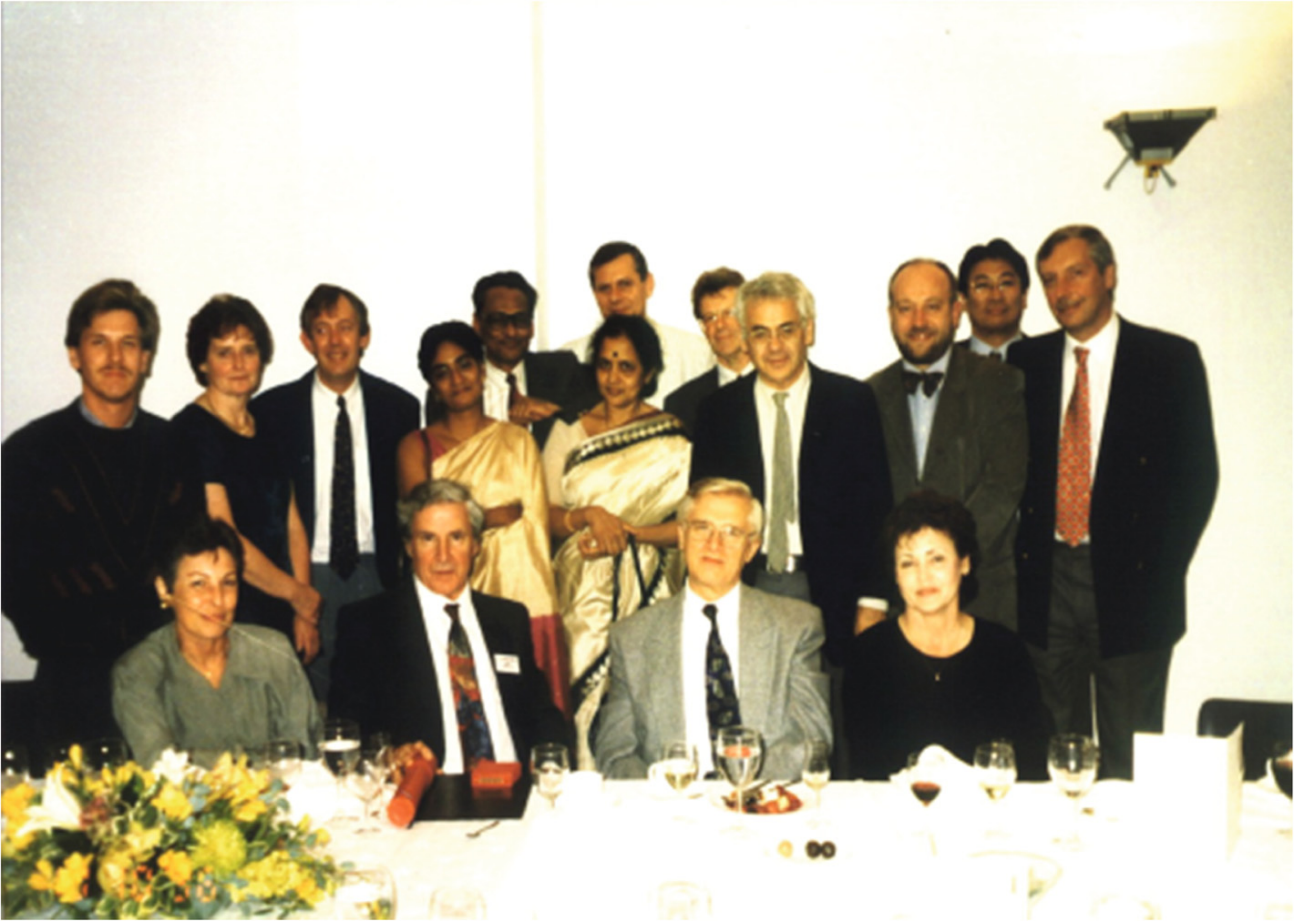
**The First International Cough Symposium post-dinner picture taken at the Royal Society of Arts in London in June 1996.** John Widdicombe is surrounded by ‘cough’ colleagues from India, Japan, Slovakia, UK and US.

**Figure 2:**
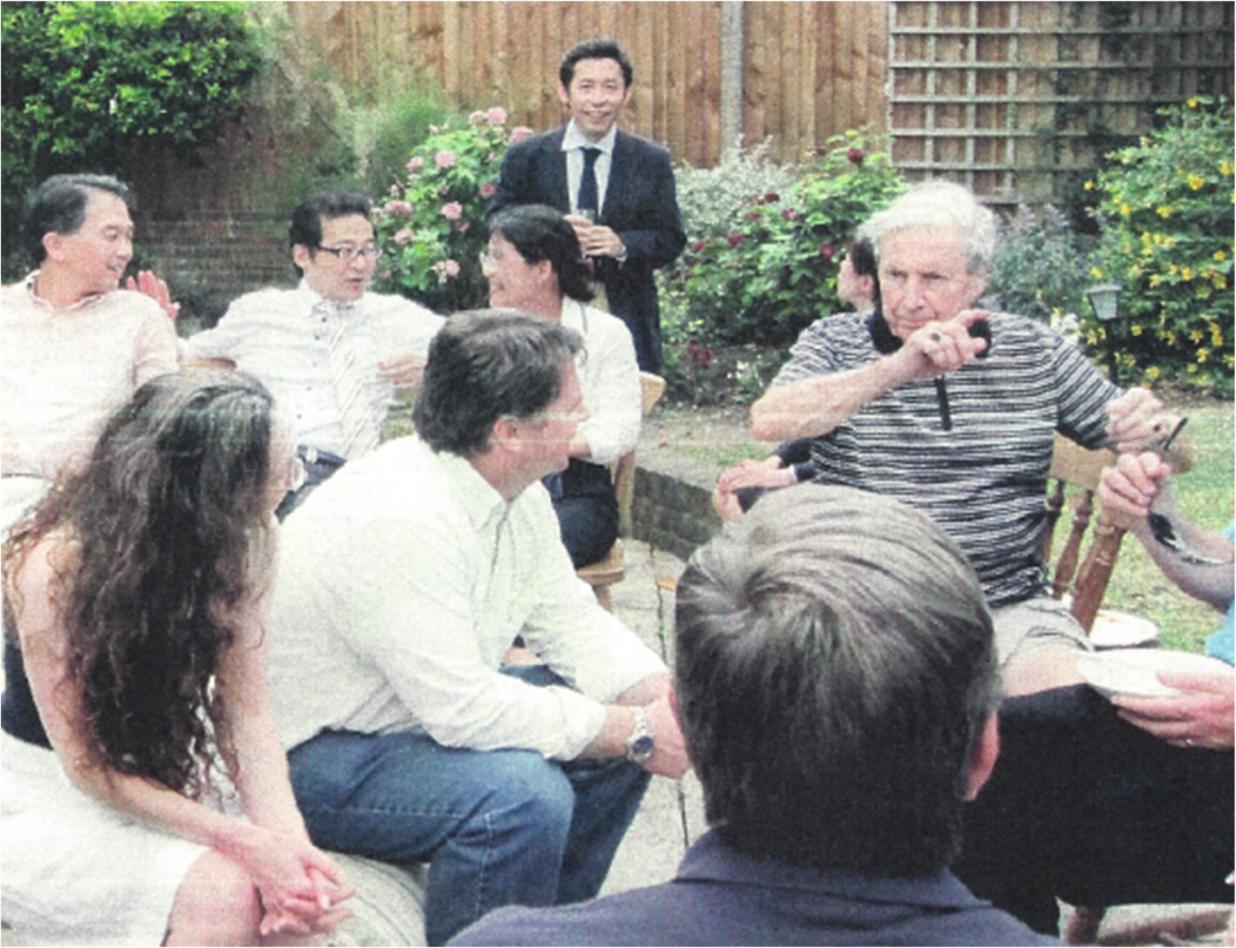
**The Sixth International Cough Symposium post-conference lunch at John Widdicombe’s home.** He is discussing with colleagues from Japan and US.

The Seventh International Cough Symposium was held in memory of its founder, John Widdicombe, in London on 5-7^th^ July 2012. The field of cough has advanced over the last 10 years at a tremendous pace thanks in part to the enthusiasm of John Widdicombe in initiating and championing these 2-yearly symposia. This was the first regular series of meeting of its kind. It all started in 1996 with the First Symposium (Figure 1). It was uniquely focused on all aspects of Cough, allowing scientists and clinicians who had a primary interest in cough to meet and share new data. The discussion went beyond the confines of the symposium and spilled into the lunches and dinners, initiating the start of many friendships and ensuring collaborations that is still endured and renewed with each successive meeting. The post-symposium afternoon lunch at the Widdicombe household in Wimbledon was another hallmark of these meetings (Figure 2). Another characteristic is the institution of publications of the Symposia with contributions from all participating speakers in Pulmonary Pharmacology Therapeutics thanks to the support of Clive Page. These publications represent a unique set of state-of-the-art reviews of contemporary cough research and thinking.

The Seventh Symposium was held in the same spirit as the preceding six. John had participated in the planning of each of the seven symposia with great enthusiasm as usual and with the Seventh Symposium, this was no exception despite having undergone knee replacement surgery and the effect of advancing years. We planned the Seventh Symposium to be held in a larger lecture theatre on the main Imperial College campus. John came over to visit the facilities soon after having undergone knee surgery! Having laid down the main Symposia for the Seventh Symposium, he unfortunately did not live to see it through. The Seventh Symposium was held in memory of John, with the first John Widdicombe lecture delivered by Brad Undem of John Hopkins on John’s contribution to Respiratory Physiology on *Vagal neurobiology of the airways*, a lecture attended by John’s wife, Margaret, and by his close family members. There were reminiscences from Jay Nadel and Giovanni Fontana. A second John Widdicombe lecture was delivered by Nanshan Zhong from Guangzhou, China, on the problem of Cough in China.

Tributes have already been written [1,2]. This article will focus on John’s contribution to Respiratory Physiology and Cough, to which he remained fervently devoted to. This contribution consists of three separate pieces from Fan Chung, Jay Nadel and Giovanni Fontana that describe our own personal experience of John.

### Appreciation from Fan Chung

#### Early encounters with John

John was a reviewer of an early piece of work that I and my supervisor, Phillip Snashall, had submitted on the role of the vagus nerves in controlling lung volumes [3]. John was the one reviewer who revealed his identity. John’s review was a helpful set of well-balanced pluses and minuses of the work. Another encounter was at the World Physiology Congress in Helsinki in the summer of 1989 where I had not only attended a session on Bronchial Blood Flow which John presided on, but also the honour of participating at a sauna party organised by Lauri Laitinen from Helsinki who had worked in his lab. I got to know the work of John, Lauri and Annika Laitinen on the bronchial circulation [4-7]. Further contacts came through the regular meetings of the Da Vinci Society for the study of the Bronchial Circulation, of which John was one of the founding fathers along with John Butler, Adam Wanner and others. In the 1990’s, Peter Barnes and I held meetings over the years at the National Heart & Lung Institute to discuss various themes of asthma research together with John and Abe Guz. These meetings were always a very good exchange of ideas centred on the airway smooth muscle, airway nerves and the bronchial circulation. It also was an occasion to reminisce on the early studies that Abe Guz and John Widdicombe did on the vagus nerves, particularly one in which John was a subject undergoing anaesthetic blockade of these nerves at the base of the skull [8]. In those days, ethical issues were not the order of the day!

My next closer interaction and encounter with John started when he invited me to co-organise the Second International Cough Symposium in 2003, an invitation that I immediately accepted on the spot. It has been a pleasure to work with the pioneer and the established expert in cough receptors [9-11]. John had already retired from his Chair at St George’s Hospital but still active in organising conferences and writing papers. We must be lucky that John was not diverted by his other hobby at the time which was the physiology of dragons [12]!

#### Cough as a major interest: early years

In the 1950’s, John embarked on his first research project which was a study of rapidly-adapting receptors (RARs) in the trachea and large bronchi, that were mostly stimulated by both inflammation and deflation of the lungs. He characterised them as Aδ-myelinated fibres. John concluded that these fibres caused cough and increased breathing. In 2003, John described how he came to work on the RARs that led to the publication of his series of 3 case selective papers of his early seminal research career, which also were the basis of his doctoral thesis in Oxford [13-15]. These were the papers that launched his career, and also initiated his interest in cough. He also concluded that he would only deserve a ‘silver’ medal having been ‘beaten’ by Keller and Loeser, the German physiologists, in discovering that RARs could induce cough [16]. Such is the modesty and fairness of John as we have always come to know. Years later, John reviewed the historical perspectives of reflexes from the lungs and airways reflecting on the explosion of our understanding of their physiology [17]. He devoted his efforts to the application of this explosion in knowledge to cough in the clinic, very much through the Symposia that he initiated and ran.

#### Later years: the cough symposia

Between 2003 and 2011, I got to know John particularly well. On the occasion of the Third Symposium, we prefaced the first publication of the symposium proceedings with a short piece on ‘Cough as a symptom’, that laid the foundation for the subsequent Cough Symposia that would focus on the relationship of cough to disease processes, different types of cough, characterisation of cough receptors and identification of peripheral and central mechanisms for cough sensitisation [18]. In a review co-authored with Giovanni Fontana on the definition of cough, he wrote: ‘Cough is usually defined as a three-phase event, although for convenience, clinicians may prefer to define it only the expiratory expulsive efforts’. He argued that cough needed to be measured with expiratory electromyograms, respiratory pressures and lung volume changes, plus cough intensity [19]. He was also interested in defining the types of cough and clinical analysis of cough which he believed would help clinicians to understand the patient’s problem clinically and visualise pathophysiological mechanisms [20]. He was also interested in the semantics of cough such as the definition of cough receptors, cough reflexes, cough in the clinic and cough sounds, and used much of his personal experience of his own cough to approach these issues. These have laid the foundation of clinical research into cough, which is an actively blossoming field having attracted many investigative clinical scientists. Part of this foundation also came from John’s long-standing collaboration with the respiratory cough group in Slovakia previously led by Professor Korpas, who was a frequent attender of these Symposia.

John organised in the 2008 Symposium a workshop on the influence of exercise speech and music on cough. These were examples of how cough could be modulated by reflexes from the airways or at a cortical level [21]. He suggested that the effects of exercise, speech, and music on cough reflexes be further explored. He had published at the same time that physiological and pathophysiological down-regulation of voluntary and reflex cough but noted that the nervous mechanisms by which these down-regulations worked had not been better studied and he had also advocated more research into this area [22]. Later, using functional brain imaging, Stuart Mazzone and others described distinct neural activation pathways relating to the network components that control voluntary cough, cough suppression and the urge-to-cough [23]. Indeed, techniques based on suppression of cough reflexes are now used as supportive measures in the treatment of cough [24].

In the 2010 Symposium, this theme was continued with another Workshop on ‘tuning the cough centre’, with the most prominent cough scientists in the field. The conclusion from the workshop was that ‘cough is not a stereotyped output from the medullary ‘cough center’, but that its pattern and strength depend on many afferent inputs acting on the ‘cough center’. One of the greatest strengths of John was his ability to provide a synthesis of the problem in such pithy accurate language. It would be fair to say that the current concept of cough hypersensitivity syndrome [25] that was discussed at the 2010 Symposium was one that emanated from the workshops John had organised.

John was interested in ways of preventing aspiration pneumonia following strokes. He pointed out the expiration reflex from the larynx leading to glottis closure could be protective in this circumstance, while the presence of an initial inspiratory phase, an essential component of both voluntary and reflex cough, would favour aspiration. With his collaborators Robert Stephens and Robert Addington, he demonstrated that retention of the expiratory reflex after stroke but less so of the voluntary cough after a stroke [26]. At the Sixth Symposium in 2010, John wrote about the assessment of aspiration risk after stroke with different types of cough, voluntary cough, reflex cough, laryngeal respiratory reflex and cough on swallow, with a review of how each could be assessed and valued [27].

In his collaboration with Giovanni Fontana, they described the deflation cough as a special cough caused by deep lung deflation, an example of a ‘positive feedback reflex response’, similar to the Head paradoxical reflex that John was very much an expert on [28,29]. They reported that all patients with deflation cough present symptoms of gastroesophageal acid reflux, possibly evoked by the efforts of lung emptying. This final work of John represents a full circle again of John’s first foray into lung reflexes to the description of another reflex of importance in cough.

#### John Widdicombe’s legacy

John leaves an important legacy. He was interested in all aspects of cough, and collaborated with many groups. He had time for the younger scientists and clinicians. He seeded ideas through the Cough Symposia, many forming the basis of current research and dogma. But dogmatic he was not, and always ready to change his thinking according to new experimental data. The International Cough Symposia that John Initiated will continue in the format he conceived it. It will remain the forum for exchange of the latest information, research and thoughts about cough, and the search for better treatments for chronic cough, as John would have wished. Future symposia will continue to remember John’s significant contribution and impact to Cough with the setting up of a John Widdicombe lecture at its 2-yearly meeting.

Many thanks, John (and I can hear him mutter: ‘that’s not necessary’).

### Appreciation from Jay Nadel

I came to the Cardiovascular Research Institute at the University of California, San Francisco in 1958 to perform pulmonary research under Dr. Julius Comroe, and I initiated studies on airway smooth muscle. In 1960, John Widdicombe came to the CVRI from Oxford as a Visiting Scientist. John’s research studies at Oxford began in 1950 under Jeffrey Dawes, studying cough and respiratory reflexes. Little was known about airway smooth muscle regulation, so together we examined the autonomic nervous regulation of bronchomotor tone. Simulation of the larynx was known to be a potent stimulus for cough (an area of John’s expertise), so we examined the effect of laryngeal stimulation on bronchomotor tone. We showed that mechanical stimulation of the larynx activated afferent nervous pathways in the vagus and subsequently increased parasympathetic nervous activity in vagal efferent nerves, causing airway smooth muscle contraction via muscarinic nervous activity [30]. I was surprised by the severity of the bronchospasm induced by a vagal reflex. The observation was novel but what is much more interesting in retrospect is that so many subsequent investigations were stimulated by the larynx study. Because of the subsequent impact of this early study with John, I will describe some of the subsequent studies that evolved from these experiments.

#### Effects of inhaled pollutants on airways

The early 1960s were times of devastating air pollution, especially in England, known as the “London Fog.” In our first studies on inhaled air pollutants, we delivered presumably inert carbon dust into the airways of anesthetized cats, and we measured airflow resistance (a measure of airway narrowing). We also examined action potentials in vagal efferent nerve fibers to the lungs. Dust increased airflow resistance, an effect that was inhibited by blockade of the vagus nerves. Dust also resulted in rapidly adapting nervous discharges, interpreted as coming from “cough” or irritant receptors in the epithelium. We found that carbon dust inhalation caused bronchoconstriction in healthy humans and in anesthetized cats [31]. Our results implicated vagal afferent pathways and vagal efferent pathways in dust-induced effects. From these findings we concluded that inhaled and deposited particulates can cause bronchospasm both in animals and in healthy humans and that autonomic pathways play a significant role in these responses.

After we showed that mechanical irritation caused bronchospasm in healthy humans and animals (larynx, dust manuscripts), we hypothesized that chemically irritating pollutants such as sulfur dioxide could also play important roles as bronchoconstricting agents. In 1965 we examined the effect of inhalation of sulfur dioxide (SO_2_) on airway resistance. Inhaled SO_2_ cause bronchoconstriction in healthy subjects and in anesthetized cats, an effect that was blocked by muscarinic antagonists [32]. We found that asthmatic subjects had a lower threshold and greater bronchial responsiveness to SO_2_[33], suggesting that air pollutants such as SO_2_ could trigger attacks of airway obstruction in asthmatics, because of their increased bronchomotor responsiveness. Subsequently, we reported that experimental exposure of human subjects to ozone also caused bronchoconstriction [34]. Although these studies were opposed by oil and gas producers, they became the basis of the original California Air Pollution Regulations, which also became the US National Standards.

#### Treatment of airway smooth muscle contraction by muscarinic antagonists in COPD

In the latter half of the Twentieth century, beta adrenergic agonists were well recognized as bronchodilators and were used clinically to relax contracted airway smooth muscle. I was invited by Boehringer-Ingelheim pharmaceutical company to provide an overview of the status of therapies of obstructive airway diseases. Remembering my early work with Dr. Widdicombe two decades before, I suggested that muscarinic antagonists could have an important role in preventing bronchospasm. This resulted in the development of Atrovent as an effective bronchodilator in COPD. Many pulmonary experts were opposed to the idea. The experimental work with Widdicombe convinced me that the muscarinic mechanism must be explored. In the editorial “Adoration of the Vagi” published in the New England Journal of Medicine [35], I suggested that knowledge of the “sociology” of the various lung cells and their actions and interactions were likely to provide new insights into the therapy of bronchospastic disease. The outcome -- anticholinergic therapy -- has become a mainstay of bronchospastic therapy in COPD. The idea is a direct result of the studies instituted together with John Widdicombe.

In this memorial, I have described a few of our early “studies” that we performed together. I did this to give examples of John’s impact on the work of other investigators (in this case mine!). Why is John so special? First, he is a problem solver. He recognizes important questions and how to solve them. Second, he understands the importance of language in science, and he is meticulous in his use of language to communicate ideas. Third, John recognizes the importance of sharing ideas. Fourth, he is immensely generous and modest. John Widdicombe contributed greatly to his many colleagues and friends. He is an unusually bright and passionate man. He has left an indelible mark on those whose path in life crossed his. Thank you, John.

### Appreciation from Giovanni Fontana

The first time I saw John Widdicombe – but had no chance to meet him – was as early as 1978 (or perhaps 1979), when he gave an outstanding lecture in Milan mainly focusing on the functional anatomy of the airways. He presented fascinating electron microscope scans of the airway mucosa. To me, it was kind of an imprinting: since then, John’s name was engraved in my memory and his work on neurophysiology of the airways reached and settled at the top of my preference list. But the first time I had a chance to meet him was only several years later and, the tricks of fortune!- the occasion was the same as that mentioned above by Fan: the physiology meeting in Helsinki. I saw John having a break somewhere in the congress hall and I could not resist inviting him to comment on my poster presentation. He was kindest to me, but unfortunately this initial approach was not followed by further contacts until early 2001 when, with much surprise, John invited me to participate in the Second London Cough Symposium: that was the beginning of a profound friendship and exciting co-operation.

John had a genuine interest in human physiology, an interest he probably had no time to cultivate during his academic career when he concentrated his energies, efforts and talents on experimental respiratory neurophysiology. In this respect, his retirement in 1992 was an opportunity: freed from need of keeping up with pure physiology, he got involved more and more deeply into human studies. For all those who had the privilege to co-work with John during his later years, his research inquisitiveness and his extraordinary qualities as a mentor in the field of clinical respiratory physiology were the ideal complement to the innovative ideas he promoted in research. He used to tease himself by saying that “since I can no longer do research, I love interfering in that done by others!” or “it is far too easy to plan other people’s research!”. But he actually loved to be involved and the nicest aspect was that he hardly realised that any of his contributions was exceptional.

The energy John devoted to cough research in the last two decades of his life is impressive. He developed a special interest in cough plasticity and wrote an outstanding review on the topic [22]. This piece of work remains a source of inspiration and a guiding light and for those who wish to investigate the physiological conditions or disease states commonly associated with cough downregulation. It was also the background to some of the most exciting experiments to which John and the cough people in Florence cooperated. I will try to describe these contributions of John’s as little scientifically as I can.

#### Downregulation of cough in humans

I once received this multi-recipient e-mail from John:

*Dear friend, some months ago I e-mailed most of you to ask if you knew anything about reflex and voluntary cough in CCHS (Congenital central hypoventilation syndrome) (Ondine’s curse). Thank you for your replies, which were interesting. I asked because I was preparing a review on cough in CNS disease. I could find much on cough in stroke, Parkinson's disease, coma, anaesthesia and sleep, but nothing on CCHS (Congenital central hypoventilation syndrome)…… I expected voluntary cough would be present, but that reflex cough might be absent. Of the replies, only Steve Shea had published: a paper showing that cough due to distilled water inhalation was weak or absent in CCHS (Congenital central hypoventilation syndrome)*[36]*. He thought this might be due to accommodation of the cough reflex by chronic disease, but I am not sure of this since many patients with chronic lung disease or with intubation go on coughing for years. Debra Weese-Mayer thought that patients were unresponsive to intubation, a view shared by Marianne Schlaefke. I think it would make a nice research project, possibly with a practical outcome. Take patients with CCHS (Congenital central hypoventilation syndrome) (and controls) and measure/observe:*

1. *Can they cough voluntarily, and if so is the power ‘normal’?*

2. *Do they respond to a tussigenic aerosol……, and if so is the threshold concentration and strength of response ‘normal’?*

3. *Do they have a normal sensation and ‘urge-to-cough’ with a tussigenic aerosol?*

4. *Do they have a ‘normal’ cough in response to* e.g. *airway infection and intubation?*

Controls would be needed.

Practical outcome: non-reflex coughers should be watched for aspiration pneumonia, as with stroke patients. (Incidentally, I don’t know if anyone has shown if the cough reflex sensitivity is different in children compared with adults.) Observations should be objective, although patient questionnaires would also be useful. Any takers? I would be happy to be involved in planning, but could not be directly involved.

Best wishes. John.

Although not “directly involved”, the contribution John gave to our paper on cough in central congenital hypoventilation [37] went well beyond the study planning: he offered us continuous assistance and guidance throughout the experiments and manuscript preparation. We managed to answer virtually all his original questions and, most importantly, participating in that study was an amazing experience for all co-authors.

The cough people in Florence were lucky enough to co-work with John in another interesting study on cough plasticity: the effect of steady state exercise and voluntary isocapnic hyperpnoea on cough threshold, force and sensation [38]. Again, from John’s email:

Dear Giovanni,

I hope you are having a wonderful holiday. I am chasing my tail……….. Let’s be serious: your single control result is wonderful and fascinating. You can’t lose.

1. It is hyperventilation and not exercise that increases the threshold to cough. Great!

2. Urge-to-cough goes up in exercise and up in hyperpnoea, in spite of the decrease in cough threshold. However the mean UTC goes down in exercise. The mind boggles!…………………. You have also K.O.’d me………. ‘One swallow does not make a summer’, but you may be on the verge of a great discovery………………. I agree with your interpretation……… But urge-to-cough is perplexing. I await confirmation or rebuttal.

Love and best wishes. John.

So, John was continuously providing us with ideas, criticism and encouragement. It was a pleasure to hear from him and learn from his knowledge, lucidity and sense of humour.

We have had several other opportunities to co-work with him, of which traces can be found elsewhere in this manuscript. For all of us, knowing that John was just always ready to support us was a stimulus to do and learn more and more.

The next-to-last email I received from John was as follows:

*Dear Giovanni, it was lovely meeting you, on personal grounds as well as for work……………… You have made me think about UtC* [urge to cough] *again, especially your comments on propofol, and I have done a rapid and incomplete literature search. I recently reviewed a paper on propofol and fentanyl and cough. Both these drugs are used to suppress cough during intubation, but both cause cough when given intravenously! Of course the same is true of capsaicin--it can either cause or inhibit cough depending on dose and route of admin. For propofol most of the literature is ‘unscientific’, but there are two good papers that show clearly that propofol does not inhibit cough caused by caps and CA* [citric acid]. *I could find no papers on UtC, but did not look very hard. But the implication, to be tested, is that you don’t need UtC to cause or augment a reflex cough. Is this a new thought?..................With voluntary cough you have no UtC. You do as you are told. If you burn your finger, you don’t need an urge to withdraw to supplement the actual withdrawal reflex. No time now to follow this up--just about to pack for France………….*

Love and best wishes. John.

“Any takers?”

## Competing interests

The authors declare that they have no competing interests.

## Authors’ contributions

KFC, JAN and GF all contributed to writing the Introduction, and each wrote their particular sections. All authors read and approved the final manuscript.
